# Remote Symptom Monitoring With Ecological Momentary Computerized Adaptive Testing: Pilot Cohort Study of a Platform for Frequent, Low-Burden, and Personalized Patient-Reported Outcome Measures

**DOI:** 10.2196/47179

**Published:** 2023-09-14

**Authors:** Conrad Harrison, Ryan Trickett, Justin Wormald, Thomas Dobbs, Przemysław Lis, Vesselin Popov, David J Beard, Jeremy Rodrigues

**Affiliations:** 1 Surgical Intervention Trials Unit Nuffield Department of Orthopaedics, Rheumatology and Musculoskeletal Sciences University of Oxford Oxford United Kingdom; 2 Cardiff and Vale University Health Board Cardiff United Kingdom; 3 Welsh Centre for Burns and Plastic Surgery Swansea University Swansea United Kingdom; 4 The Psychometrics Centre University of Cambridge Cambridge United Kingdom; 5 Clinical Trials Unit Warwick Medical School Coventry United Kingdom; 6 Department of Plastic Surgery Stoke Mandeville Hospital Ayelsbury United Kingdom

**Keywords:** patient-reported outcome measures, ecological momentary assessment, computerized adaptive testing, EMCAT, symptom monitoring, monitoring, assessment, smartphone app, trauma, arthritis, usability, mobile phone

## Abstract

**Background:**

Remote patient-reported outcome measure (PROM) data capture can provide useful insights into research and clinical practice and deeper insights can be gained by administering assessments more frequently, for example, in ecological momentary assessment. However, frequent data collection can be limited by the burden of multiple, lengthy questionnaires. This burden can be reduced with computerized adaptive testing (CAT) algorithms that select only the most relevant items from a PROM for an individual respondent. In this paper, we propose “ecological momentary computerized adaptive testing” (EMCAT): the use of CAT algorithms to reduce PROM response burden and facilitate high-frequency data capture via a smartphone app. We develop and pilot a smartphone app for performing EMCAT using a popular hand surgery PROM.

**Objective:**

The aim of this study is to determine the feasibility of EMCAT as a system for remote PROM administration.

**Methods:**

We built the EMCAT web app using Concerto, an open-source CAT platform maintained by the Psychometrics Centre, University of Cambridge, and hosted it on an Amazon Web Service cloud server. The platform is compatible with any questionnaire that has been parameterized with item response theory or Rasch measurement theory. For this study, the PROM we chose was the patient evaluation measure, which is commonly used in hand surgery. CAT algorithms were built using item response theory models derived from UK Hand Registry data. In the pilot study, we enrolled 40 patients with hand trauma or thumb-base arthritis, across 2 sites, between July 13, 2022, and September 14, 2022. We monitored their symptoms with the patient evaluation measure, via EMCAT, over a 12-week period. Patients were assessed thrice weekly, once daily, or thrice daily. We additionally administered full-length PROM assessments at 0, 6, and 12 weeks, and the User Engagement Scale at 12 weeks.

**Results:**

The use of EMCAT significantly reduced the length of the PROM (median 2 vs 11 items) and the time taken to complete it (median 8.8 seconds vs 1 minute 14 seconds). Very similar scores were obtained when EMCAT was administered concurrently with the full-length PROM, with a mean error of <0.01 on a logit (*z* score) scale. The median response rate in the daily assessment group was 93%. The median perceived usability score of the User Engagement Scale was 4.0 (maximum possible score 5.0).

**Conclusions:**

EMCAT reduces the burden of PROM assessments, enabling acceptable high-frequency, remote PROM data capture. This has potential applications in both research and clinical practice. In research, EMCAT could be used to study temporal variations in symptom severity, for example, recovery trajectories after surgery. In clinical practice, EMCAT could be used to monitor patients remotely, prompting early intervention if a patient’s symptom trajectory causes clinical concern.

**Trial Registration:**

ISRCTN 19841416; https://www.isrctn.com/ISRCTN19841416

## Introduction

Patient-reported outcome measures (PROMs) are questionnaires that can quantify the severity of a disease, or the impact of its treatment, from the patient’s perspective [1,2]. PROMs are frequently used both as primary outcome measures in research studies and as adjuncts to clinical care [3-8]. PROM use has been associated with improvements in quality-of-life outcomes and patient experience, more sensitive detection of quality-of-life issues, faster detection of clinical deterioration and disease recurrence, higher referral rates, and more efficient consultations [4,6,9-15]. In lung cancer follow-up, the survival advantage of remote PROM monitoring versus routine care has been great enough to mandate the early termination of a multicenter randomized controlled trial [16,17].

Often, PROMs are administered at specific, infrequent, time points. This infrequent cross-sectional sampling may fail to capture temporal fluctuations in symptom severity and introduce recall bias when patients are asked to remember how their health has been over a period of time. A solution to this is ecological momentary assessment (EMA) [18-20]. In EMA, patients complete serial PROM assessments in their natural (ecological) surroundings to report on their health state at the time of the assessment (momentarily). Administering PROMs in this way is believed to reduce recall bias [21]. EMA has clear applications in both clinical research and care, but its usefulness and uptake have been limited by the response burden it causes both to patients and administrators. Overburdening patients with multiple, lengthy questionnaires lead to response fatigue, poor engagement, and missing data [22,23].

Computerized adaptive testing (CAT) describes the use of algorithms to shorten and personalize assessments such as PROMs [24-28]. In CAT, psychometric models are used to map the responses to each item in a questionnaire onto continuous scales that reflect the measured construct. After the response to a single item, a person’s score can be estimated, and the next most useful remaining item can then be selected for that person, based on their estimated score. The CAT algorithm repeats this process, estimating the person’s score with increasing precision, until a prespecified stopping rule is met, for example, after a certain number of items or after reaching a measurement precision threshold. This is similar to a doctor taking a focused history—if we know a patient has difficulty walking 100 m, we do not need to ask whether they have difficulty walking a mile; instead, we ask whether they have difficulty getting around their home. By selecting only the most relevant items for an individual, CAT can produce scores that are very similar to full-length PROM scores from a fraction of items in the PROM [24,26,28,29]. By reducing the burden of individual assessments with CAT, we may be able to deliver higher-frequency and lower-burden EMA with better acceptability to respondents, clinicians, and trialists. We have termed this concept ecological momentary computerized adaptive testing (EMCAT).

We describe the development of a free platform that can administer any PROM that has been validated with modern psychometric techniques through EMCAT. We demonstrate the feasibility of the platform for use in research or clinical practice in 2 common conditions: hand trauma (which accounts for up to 30% of Emergency Department attendances) [30] and thumb-base osteoarthritis (TBOA, present in 8% to 12% of the general population) [31]. We suggest that our platform may provide metrological advantages over existing techniques, including contemporary methods for quantifying measurement errors at the individual respondent level.

## Methods

### The EMCAT Platform

The EMCAT progressive web app has been built using Concerto, an open-source CAT development platform maintained by the University of Cambridge Psychometrics Centre [32]. It features an HTML interface, MySQL databasing system, and a highly versatile R-based back end, with a large proportion of its functionality based on Phil Chalmers’ *mirtCAT* R package [33]. It is free to use. During this study, we hosted the app on a secure Amazon Web Service cloud server (one instance per study site).

The app can administer CAT assessments for any PROM that has been validated to modern psychometric standards (specifically, any PROM for which item response theory or Rasch measurement theory model parameters are available). Items from the PROM, together with model parameters, are uploaded to the platform as a CSV file.

The platform allows users to choose from a range of score estimators and item selection criteria when deploying their CAT assessments. Stopping rules can be based on SE of measurement thresholds, a prespecified number of items, or a combination thereof. For Bayesian estimators and item selection criteria, users are able to automatically set the new assessment prior to the posterior distribution of the previous (completed) assessment. This means that the first CAT item can be chosen for each respondent based on their most recent assessment score. This approach, which we describe as using a dynamic prior, has the potential to improve assessment efficiency and may improve the content validity coverage of the assessment by varying the first item used for an individual as their symptoms change over time.

Assessments are prescheduled for either individuals or groups of individuals, and respondents can be prompted to complete an assessment either by email, push notification (Android users), or both. The app is compatible with smartphones, personal computers, laptops, and tablets. Currently, Apple products do not support progressive web app push notifications. The email alert system, which is managed through Mailgun via an application programming interface, provides a unique URL to the respondent’s assessment, which is opened in a normal web browser.

During this study, we presented respondents with a time series display of their scores over the trial period, each time they completed an assessment. This feature can be adapted to fit the clinical context. Scores are available on the researcher- or clinician-facing side of the platform, in real time.

### Patient Selection

We recruited a sample of consecutive patients undergoing care for recent hand trauma or TBOA at Buckinghamshire Healthcare National Health Service Trust and the Cardiff and Vale University Health Board between July 13, 2022, and September 14, 2022. Patients were followed up for a 12-week period using the EMCAT platform.

We included patients aged 18 years or older who were willing and able to provide informed consent, and download and use the EMCAT app on a personal smartphone. Patients with a cognitive or communicative barrier to study engagement were excluded. Patients were screened for inclusion by clinicians involved in their usual care during routine clinic appointments and where necessary, assistance in downloading the smartphone app was provided by research nurses. All patients provided written and informed consent to take part in this study.

Prior to recruitment, the study was registered in the ISRCTN registry (ISRCTN19841416) and the National Institute for Health Care Research (NIHR) Central Portfolio Management System (51091), sponsored by the University of Oxford Research Governance, Ethics and Assurance team (PID15769), and approved by the Health Research Authority and Health and Care Research Wales.

### Questionnaires and Scheduling Regimes

The principal measure in this study was the CAT version of the patient evaluation measure (PEM; Part 2) [34]. The PROM contains 11 items measuring hand function, which are each scored with 7 response categories. We scored the PEM using item response theory parameters that were derived from the UK Hand Registry and published previously [29]. This method produces continuous *z* scores on a logit scale, mostly ranging from –2 to +2, where a higher score indicates a greater degree of symptom severity and 0 represents the mean of the calibration sample.

The CAT was programmed to estimate scores using an expected “a posteriori” approach. Items were selected using a minimum expected posterior variance item selection criterion, and the assessment terminated once the SE of measurement dropped below 0.3. This level of precision approximately equates to a marginal reliability of 90% and is consistent with other popular CAT platforms [26,35,36].

For each patient, the first item in the first assessment was selected based on a standard normal prior distribution. In subsequent assessments, we used the dynamic prior feature to update the starting prior.

Patients were allocated to receive EMCAT assessments either thrice weekly, daily, or thrice daily. Allocations were made based on consecutive study number and specified prior to recruitment. Patients were prompted to complete each assessment by email, and Android users additionally received a push notification for each assessment. There was no time limit to completing an assessment, and all responses were time-stamped. The face validity of the PEM and the appropriateness of these scheduling regimes were confirmed with patient and public involvement (PPI) partners from the British Society for Surgery of the Hand PPI group prior to study commencement.

In addition to the CAT version of the PEM, we also administered the full-length PEM to each patient at 0, 6, and 12 weeks, and the 30-item User Engagement Scale (UES) at 12 weeks. All assessments were administered via the EMCAT platform. The UES contains 4 independently scored subscales measuring perceived usability (8 items), aesthetic appeal (5 items), focused attention (7 items), and reward factor (a combination of endurability, novelty, and felt involvement, 10 items) [37]. The most relevant UES subscale to this work (which aims to reduce response burden) is perceived usability, defined as “negative affect experienced as a result of the interaction and the degree of control and effort expended.” Each UES item contains 5 response options, with a higher score indicating a greater level of engagement. We scored each scale by calculating the mean item score, excluding response sets that were missing more than a single-item response.

### Statistical Analysis

We used descriptive statistics to present demographics, response rates, response times, the frequency each PEM item was used, and UES scores. Time series plots are illustrated for a patient with each condition.

We identified instances when the full-length PEM and its CAT counterpart had been completed by an individual on the same day. For these cases, we compared the similarity of full-length PEM scores and CAT scores by calculating mean error. We also used time-series plots to visualize full-length PEM scores with reference to EMCAT score trajectories.

A potential limitation in the way PROMs are currently used in research and clinical practice is that measurement error is seldom accounted for at the individual level. This is clearly relevant for high-stakes decision-making (eg, in clinical practice), and may be a potential source of bias for clinical trials and observational studies. Techniques for estimating and accounting for individual-level PROM measurement errors are emerging [38,39], and we have built EMCAT with these in mind. We illustrate 3 ways individual-level measurement error can be estimated with the EMCAT platform: the use of 95% credible intervals, presenting scores as probability densities based on their posterior distributions, and the use of time series smoothing (in this case, locally estimated scatterplot smoothing [LOESS] [40]).

### Ethics Approval

Ethical approval for this study was granted by the Cambridge East National Health Service Research Ethics Committee (21/EE/0261). All participants provided informed consent. Study data have been deidentified. Participants were not provided with financial compensation for their involvement in this study.

## Results

### Patient Characteristics

We recruited 40 patients across 2 sites, 20 from each site, 10 patients with TBOA, and 10 patients with hand trauma, respectively. One patient with hand trauma did not engage in any assessments and was withdrawn from the study.

The median age of included participants was 59 (range 19-83) years, with 26 participants female and 13 male. Over the 12-week study period, 12 patients were allocated to receive EMCAT assessments thrice weekly, 15 were allocated to receive daily EMCAT assessments, and 12 were allocated to receive thrice daily EMCAT assessments.

### Response Rates, Items Posed, and Response Times

Response rates were highest in patients allocated to daily assessments (median response rate 93%, range 33%-100%). Response rates were lowest in those administered thrice daily assessments (median response rate 43%, range 6%-98%). The median response rate for patients completing thrice weekly assessments was 63% (range 33%-100%).

**Figure 1:**
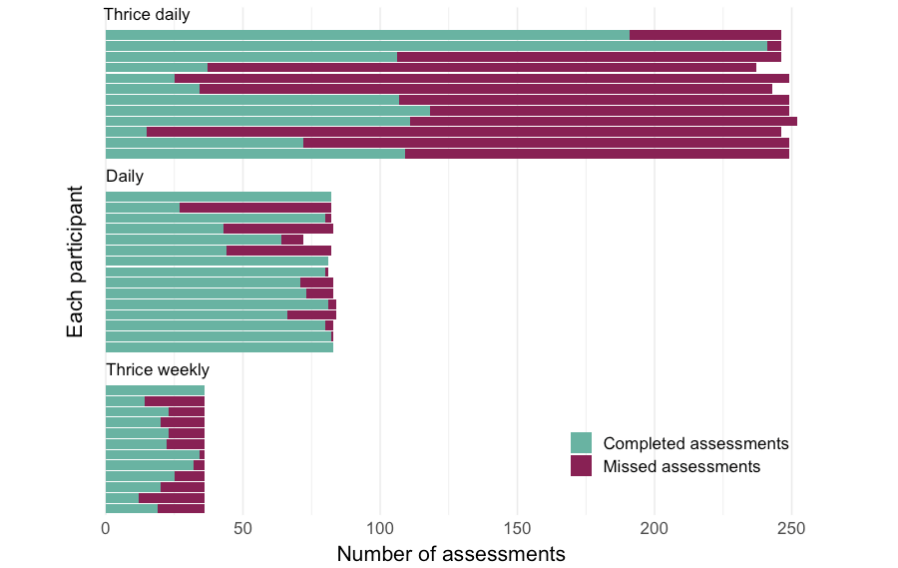
Number of ecological momentary computerized adaptive testing (EMCAT) assessments completed and missed by each participant, over the 12-week study period.

Even in patients with low response rates, the absolute number of completed assessments was high. The total number of completed EMCAT assessments over the 12-week period ranged from 12 to 241 per participant. For each participant, the number of completed and missed EMCAT assessments is presented in Figure 1.

Across all EMCAT assessments, the median number of items used from the full-length (11-item) PEM was 2 (range 1-4).

The item most commonly selected by EMCAT was item 8 (the starting item for a standard normal prior distribution, administered 2509 times during the study). This was followed by item 3 (administered 1231 times during the study). Items 1 and 2 were not selected by the algorithm at any point in the study.

The median time taken to complete each EMCAT assessment was 8.8 (IQR 5.5-14.2) seconds. This compared to a median time of 1 minute 14 seconds to complete the full-length PEM (IQR 52.5 seconds to 2 minutes 14 seconds).

**Figure 2:**
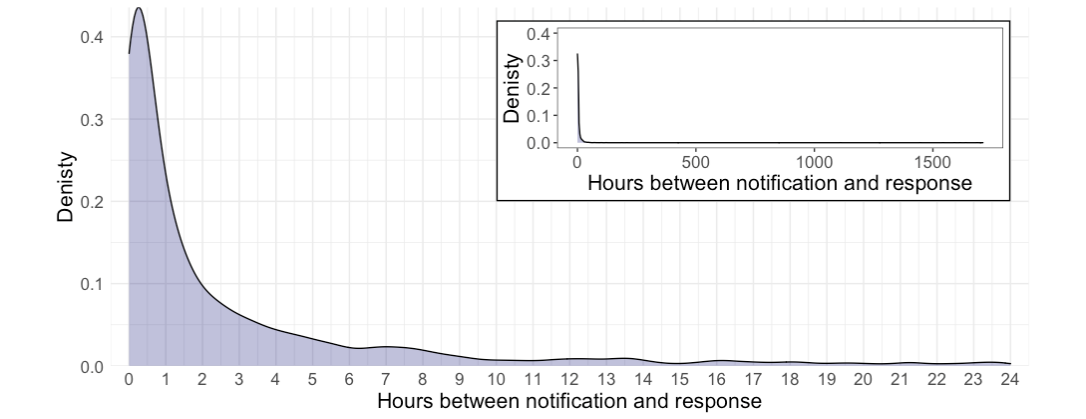
The distribution of times taken between prescheduled notification and receiving the completed assessment.

Most assessments were completed within an hour of the prescheduled notification times. The median time between notification and response was 54 minutes and 15 seconds (IQR 11 minutes 54 seconds to 3 hours 33 minutes; Figure 2).

We received responses to 74 complete response sets out of 117 full-length PEM assessments over the 12-week period, and 23 completed UES questionnaires at the end of the study. Within these 23 UES responses, there were no missing responses to items in the perceived usability or focused attention subscales. One item from the aesthetic appeal subscale was missed for 1 patient, and this response set was included in the analysis. In total, 2 response sets were excluded from the Reward Factor subscale as they missed 9 and 10 items respectively.

### Usability

Responses to the UES supported the acceptability of EMCAT. The median perceived usability score was 4.0 (range 2.9-5.0, maximum possible score 5.0). For those receiving thrice daily assessments (n=5), the median perceived usability score was 3.6 (range 2.8-3.9). For those receiving daily assessments (n=14), the median perceived usability score was 3.9 (range 3.1-4.8). For those receiving thrice-weekly assessments (n=4), the median perceived usability score was 4.1 (range 3.6-4.3). Of the 23 respondents, 21 either disagreed or strongly disagreed with the item “using the EMCAT app was taxing,” while 2 respondents (both assigned daily assessments) neither agreed nor disagreed. The median focused attention, aesthetic appeal, and reward factor scores were 2.1, 3.1, and 3.3 (ranges 1.0-3.6, 2.0-4.2, and 2.1-4.3), respectively.

### Symptom Trajectories

**Figure 3:**
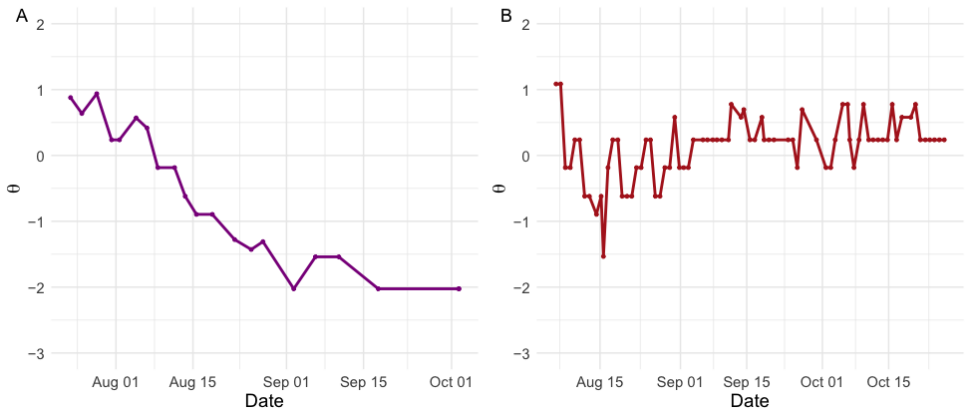
Symptom trajectories for a patient with a fractured metacarpal (A) and thumb-base osteoarthritis (B).

Figure 3 illustrates 2 symptom trajectories recorded by the EMCAT platform, with *z* scores presented on the y-axes. A higher score indicates greater symptom severity. The magenta plot (panel A) demonstrates a patient recovering from a metacarpal fracture (a common injury to one of the bones in the hand that usually has an excellent prognosis). The red plot (panel B) shows the clinical course of a patient with TBOA who received a steroid injection on August 5; their symptoms improve over a 10-day period following the injection, then return to near-baseline levels. The patient with TBOA reports greater fluctuations in symptom severity than the patient with the metacarpal fracture, consistent with the diagnoses (the symptom burden of TBOA is known to vary with activity, by time of day, and stochastically to some extent [41,42], whereas metacarpal fracture symptoms were expected to improve and resolve over the 12-week observation period [43]).

**Figure 4:**
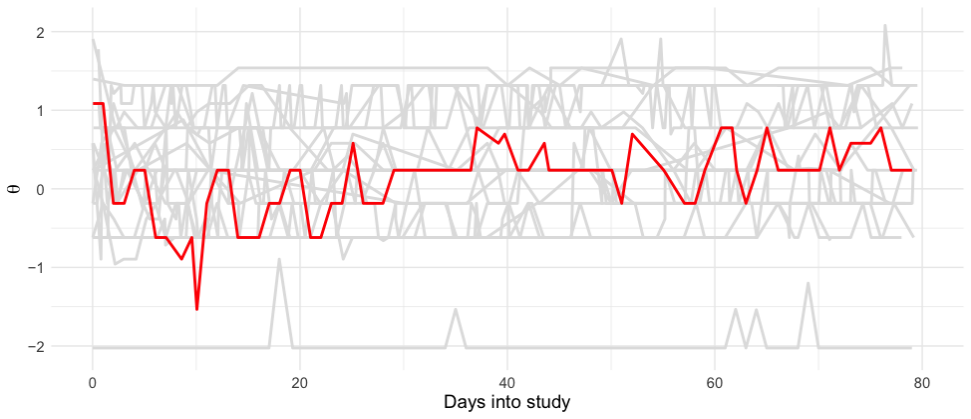
A symptom trajectory contextualized with reference to other patients. The symptom trajectory of the patient in Figure 3B is presented in red, while all other patients with thumb-base osteoarthritis (TBOA) are presented in gray. The outlier with low symptom severity is a respondent who had historical treatment and was seen as part of long-term follow-up while able to fulfill all activities of daily living.

While *z* scores are directly interpretable (a score of 0 is equivalent to the mean score of the calibration sample), it is possible to modify the EMCAT platform to present scores with further context, for example, with written feedback to the patient, or with clinically important thresholds superimposed on the graphical output. In some cases, with appropriate consent, it may be possible to contextualize a person’s trajectory with reference to other people’s trajectories. Figure 4 illustrates the clinical course of the patient in Figure 3B in relation to other patients with TBOA included in the study. Here, we can understand that the symptom trough following their injection reflects a relatively mild clinical state, compared to the other patients in the study.

**Figure 5:**
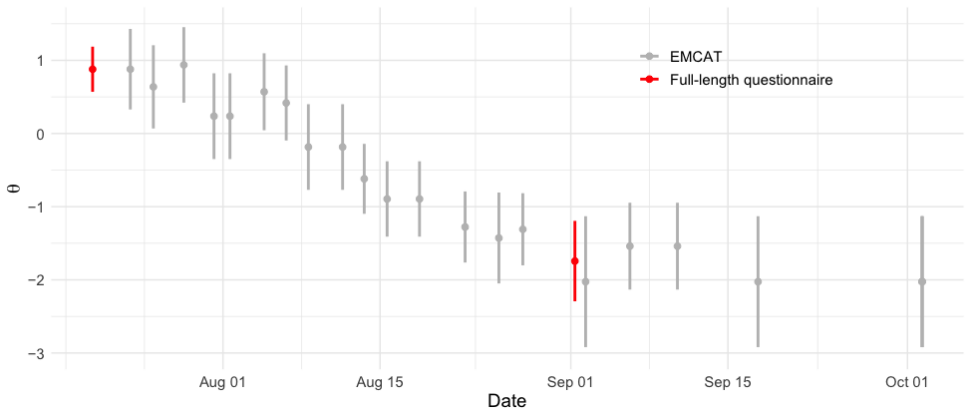
Full-length patient-reported outcome measure (PROM) and EMCAT scores. Gray points indicate symptom severity measurements made by the EMCAT algorithm and red points indicate z scores derived from the full-length questionnaire. Error bars represent 95% credible intervals, based on the normal approximation to each score’s posterior distribution. The full-length scores are consistent with the symptom trajectory as measured by EMCAT, further supporting the use of EMCAT scores as approximations to full-length PROM scores. EMCAT: ecological momentary computerized adaptive testing.

In 27 instances, patients completed the full-length PEM on the same day as an EMCAT assessment, allowing us to compare the similarities of EMCAT scores to those derived from the full-length questionnaire. The mean error between PEM scores and same-day EMCAT scores was negligible in this sample (<0.01 on the logit [*z* score] scale). Figure 5 illustrates full-length PEM scores in relation to the trajectory implied by EMCAT scores, for the patient in Figure 3A.

### Quantifying Uncertainty

**Figure 6:**
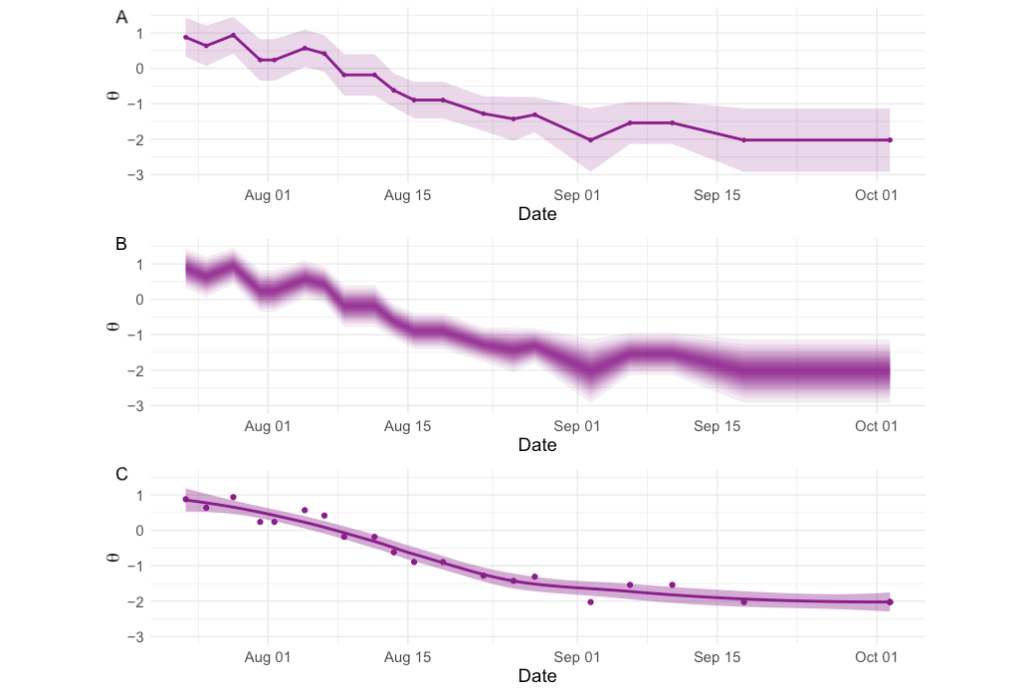
Techniques for illustrating measurement uncertainty in ecological momentary computerized adaptive testing (EMCAT) scores. Panel (A) demonstrates 95% credible intervals around each measurement, as is currently implemented in the EMCAT platform. Panel (B) extends this concept by illustrating the probability densities within these credible intervals. Darker portions of the plot are more likely to contain the patient’s true symptom level. Panel (C) provides an example of time series smoothing, where the patient’s symptom trajectory is fitted to minimize the square of residual terms locally. The shaded proportion of the figure represents a t-based approximation to the SE [40].

Currently, the platform can be set either to display score estimates alone (as in Figure 3) or with 95% credible intervals (Figure 6A), which are based on the normal approximation to either the score’s posterior distribution or likelihood function, depending on the choice of estimator. Figure 6 demonstrates 2 other potential strategies for modeling individual-level measurement uncertainty in EMCAT scores. Figure 6B illustrates the probability density for the person’s latent construct (hand symptom) level, based on their observed score (in this case, the density is based on the normal approximation to the score’s posterior distribution), and Figure 6C demonstrates the use of time series smoothing (in this case, LOESS) for estimating measurement error, which may be helpful if the patient’s symptoms are expected to follow a smoother trajectory.

In research and clinical practice, PROM scores are not typically presented with reference to potential measurement error at the individual level, although PROM scores are unlikely to perfectly reflect the level of the measured latent construct (ie, symptom severity in the mind of the patient). Research is beginning to emerge in this area [38,39,44], and illustrations of measurement uncertainty may be useful for comparing patients to each other, or to clinically relevant thresholds.

## Discussion

### Principal Findings

In this study, we have demonstrated the EMCAT platform as a tool for collecting remote, low-burden, and highly granular PROM data for both research and clinical care. We show that CAT allows the platform to produce precise PROM scores from fewer items and significantly shorter assessment times, supporting previous in silico findings [29]. Accurate prescheduling was possible, with most assessments returned within an hour of their prespecified time. The perceived usability of the platform was high, and this was reflected in high response rates for those administered daily or thrice weekly assessments. While response rates for those administered thrice daily assessments were low (43%), the absolute number of assessments completed was still high, with most participants in this group completing over 100 assessments during the 12-week period. These sampling frequencies exceed those typically reported by studies aiming to monitor patients with traditional PROMs of similar lengths to the PEM [9,17,45,46]. We believe the EMCAT platform will allow clinicians and researchers to administer EMA assessments more frequently (or with more PROM scales) than they otherwise could, with similar accuracy. This is likely to lead to deeper insights into clinical research and more responsive clinical monitoring.

The EMCAT platform is free to use. While this paper demonstrates its use with the PEM in 2 common hand conditions, we have developed it to be deliberately modular, so that the PEM can be replaced with any questionnaire that has been validated with modern test theory [47]. This includes PROM and non-PROM questionnaires, such as satisfaction surveys or even educational assessments.

The EMCAT platform may be particularly useful in medical research for studying the impact of an intervention on symptom trajectories. For example, in a trial of open versus endoscopic surgery, one might expect the long-term results to be similar if both operations resect, implant, decompress, or repair the same thing. However, the endoscopic approach might result in a faster recovery. This benefit can be difficult to observe with traditional trial sampling techniques. It would require trialists to correctly predict when the differences in PROM scores between trial arms will exist, and plan to measure the groups at that point. This is further complicated when patients follow heterogenous recovery paths. The EMCAT platform provides a clear solution to this problem. It might also be well suited to studying conditions with large temporal fluctuations in symptom severity (eg, TBOA and other inflammatory conditions, mental health problems, and metastatic cancer). In these cases, trialists may be concerned about bias that can occur when a patient is measured on a particularly “good” or “bad” day, or when they are asked to recall and average their symptoms over a period of time.

The most straightforward way to analyze EMCAT data is likely to be with mixed-effects linear models, as is common practice for trials with repeated measures [48]. However, EMCAT will also support researchers who wish to apply contemporary psychometric techniques to model measurement error, some of which are illustrated in Figure 6.

The EMCAT platform has applications in clinical practice. Remote follow-up has become increasingly popular following the COVID-19 pandemic and is of interest to public health services dealing with the financial impact of the pandemic [49,50]. The EMCAT system could be used to check whether patients are recovering from a treatment as expected, and prompt early intervention if their recovery falters. It could be used to trigger repeat interventions based on patient need (eg, the use of steroid injections to treat symptoms of TBOA). It could also be used to enhance routine clinical surveillance, for example, by detecting symptoms of cancer recurrence. The use of EMA and CAT to classify patients into those who may or may not benefit from momentary (just-in-time) interventions has been described previously under the label “just-in-time adaptive ecological momentary assessment” (JITA-EMA) [51]. Simulation studies published earlier this year demonstrate gains in classification accuracy over conventional methods when these techniques are used, with a 13-item fatigue scale, with the aim of identifying momentary fatigue states [51]. Our paper complements this work by illustrating the real-world feasibility and acceptability of such systems.

In the future, the clinical usefulness of EMCAT could increase through the incorporation of wearable sensor data, either to trigger assessment prompts or even to include as part of the psychometric modeling process (eg, step count could be used to select the most appropriate starting item for a mobility CAT assessment). Ongoing work in “tipping point” modeling may also enhance EMCAT, through the ability to automatically anticipate early clinical deterioration based on symptom trajectory, particularly in mental health contexts [52-54].

There are limitations to this study. We have evaluated EMCAT with a single questionnaire, in 2 conditions, across 2 sites, and our findings may not generalize to other settings. Our sample did include older patients and those who have difficulty using their hands, 2 potential barriers to using the EMCAT app [55], but it will be important to evaluate the platform in broader and more diverse patient groups in the future, including those with visual impairments, cognitive decline, and fatigue. We expect that the use of EMCAT will reduce the assessment burden in most patient groups, compared to traditional techniques, and this may improve research inclusivity. In this study, we allocated each patient to one assessment frequency (thrice daily, daily, or thrice weekly), rather than allocating all patients to each assessment frequency for a period of time. With this (and our small sample size) in mind, it is difficult to interpret the finding that daily assessments achieved higher response rates than thrice weekly assessments (which one might imagine are less burdensome). That said, it is plausible that daily assessments are easier to remember, and become more routine, than thrice weekly assessments. Finally, our study did not include a comparator group that was posed full-length linear assessments. This might have provided valuable evidence that EMCAT results in higher compliance than traditional EMA. These study designs should be considered in future research.

In this study, items 8 and 3 from the PEM were used more frequently than others, and one might wonder whether similar results could be achieved more simply by administering these items as a static, 2-item, short-form, instead of using CAT algorithms. This finding is explained by specific psychometric properties of the PEM questionnaire (these items provide the most measurement information at common latent construct levels [29]), and the distribution of item administration frequency will vary with other questionnaires. CAT has been shown to produce more precise score estimates than static short forms of comparable lengths [26,56], and this is likely to have been the case for respondents who administered items other than 8 and 3 in this study. Other advantages of CAT and EMCAT over static short forms are the ability to vary items posed to a respondent over time (through techniques such as content balancing or the use of EMCAT’s dynamic prior, see Methods), and the possibility of adding additional informative items to the questionnaire without increasing the burden on the respondent—a strategy known as “item banking,” popularized by the Patient-Reported Outcomes Measurement Information System (PROMIS) initiative [25]. In this study, we did not apply any exposure control to our assessments, but it is possible to do so using the EMCAT platform. For example, a randomesque item selection criterion can be used to randomly select one of the *n* most informative items to ask, rather than the most informative item [57]. Had we done this, we would have likely seen a more even distribution of items selected. Exposure control may reduce the efficiency of the assessment, in terms of precision gained per item posed, but could potentially result in more engaging assessments with a broader content validity coverage. The impact of content balancing and exposure control should be explored in future EMCAT studies.

Currently, the EMCAT platform is only able to administer CAT assessments for PROMs that have known item response theory or Rasch model parameters [47]. These parameters are a byproduct of modern PROM validation processes, but not all PROM developers or validators make these parameters openly available. Users may also wish to use EMCAT with PROMs that have not been subject to contemporary standards of construct validation. Other techniques for administering CAT assessments, such as decision trees, have shown promise for use with PROMs that have not been parameterized with modern test theory and these should be implementable in future iterations of the EMCAT platform [58,59].

In this study, we made no modifications to the PEM items (we used the 11 items in the PEM Part 2 Hand Health Profile, which are freely available in the instrument’s validation study [34]). The PEM items do not specify a recall period (eg, by asking patients to score their symptoms over the last 7 days). However, many item banks will pose a recall period, and in these cases, items would have to be modified before they could be administered momentarily. In some cases, this could impact the items’ content validity and psychometric properties. Modified items cannot be assumed to have the same IRT parameters as the originals, and this limits the number of PROMs that could be feasibly administered via EMCAT. As EMA increases in popularity, it is likely that more item banks will be developed for the purpose of momentary assessment. This carries additional challenges for assessment developers, as EMA items are administered far more frequently than typical PROM items, and ideally, they should demonstrate stable psychometric properties over time. In the context of CAT and IRT, this would mean model parameters that are invariant over time, and across all participants (cross-level noninvariance) [51]. The extent to which item properties change between administration times (parameter drift), and whether this happens uniformly between individuals or subgroups, should be a focus of future research.

### Conclusions

The EMCAT app is a novel system for longitudinal PROM data capture, which has demonstrated a high level of acceptability and feasibility. By reducing the assessment burden, EMCAT will permit high-frequency PROM assessment in both research and clinical practice.

## Abbreviations

CAT: computerized adaptive testing
EMA: ecological momentary assessment
EMCAT: ecological momentary computerized adaptive testing
JITA-EMA: just-in-time adaptive ecological momentary assessment
LOESS: locally estimated scatterplot smoothing
NIHR: National Institute for Health Care Research
PEM: patient evaluation measure
PPI: patient and public involvement
PROM: patient-reported outcome measure
PROMIS: Patient-Reported Outcomes Measurement Information System
TBOA: thumb-base osteoarthritis
UES: User Engagement Scale

## Data Availability

The data sets generated and analyzed during this study are available from the corresponding author on reasonable request.
